# Does vacuum mixing affect diameter shrinkage of a PMMA cement mantle during in vitro cemented acetabulum implantation?

**DOI:** 10.1177/0954411920964023

**Published:** 2020-10-15

**Authors:** Alexander T Boote, Robert JA Bigsby, David J Deehan, Kenneth S Rankin, David C Swailes, Philip J Hyde

**Affiliations:** 1 Newcastle University, Newcastle upon Tyne, UK; 2 Zimmer Biomet, Bridgend, UK; 3 Freeman Hospital, Newcastle upon Tyne, UK

**Keywords:** Implants/prosthetics, orthopaedic materials, biomedical cements, hip protheses, joint prosthetics

## Abstract

Radiolucent lines on immediate postoperative cemented acetabular component radiographs between the PMMA bone cement mantle and bone are an indicator of an increased risk of early loosening. The cause of these lines has yet to be identified. Thermal and chemical necrosis, fluid interposition and cement shrinkage have all been suggested in the literature. The aim of the study reported here was to take an engineering approach – eliminating confounding variables present during surgery – to quantify the size of the interstice created by cement shrinkage when a 50 mm diameter flanged acetabular cup is implanted in a model acetabulum with a 52 mm hemispherical bore under controlled conditions using vacuum and non-vacuum mixed cement. Irrespective of the mixing method used, a significant interstice was created between the bone cement and the mock acetabulum. When the cement was mixed under vacuum the interstice created between the mock acetabulum and the cement mantle was 0.60 mm ± 0.09 mm; when the cement was mixed under non-vacuum conditions the interstice created was 0.39 mm ± 0.15 mm. Possible explanations for radiolucent lines are discussed.

## Introduction

Cemented total hip arthroplasty (THR), according to NJR statistics, has the lowest rate of revision at all time points after surgery.^1^ However, some acetabular cups still fail, aseptic loosening being the most common cause.^1^ In cases where the implant fails after a long time period, the cup and cement mantle becomes loose due to osteolysis causing a resorption of bone around the cement mantle; this is a slow process that occurs over many years.^2^ Wear debris from the articulating surfaces of the implant migrates into the interface between bone and cement, this results in an adverse reaction which triggers resorption of bone. This, in turn, creates a layer of soft tissue which can be seen on radiographs as a radiolucent line.^2^ However, this long-term bone resorption process does not explain reports of radiolucent lines on immediate post-operative radiographs around the cement mantle of acetabular cups.^3–8^ Authors have suggested these may develop due to thermal necrosis,^9–17^ chemical necrosis,^15, 16,18,19^ fluid imposition,^20–24^ and cement shrinkage.^11,14–16,25,26^

Most bone cements used for acetabular cup fixation are based on PMMA (polymethyl-methacrylate). The cement comes as two components: a powder, which is primarily ground PMMA and a liquid which is primarily MMA monomer. When the two components are mixed a polymerisation reaction starts which continues until full cure and rigidification.^27^ Polymerisation results in an increase in molecular density and therefore volume shrinkage. The reaction is exothermic so the cement mantle will generate and expel heat; this will cause thermal expansion of the cement mantle during polymerisation and subsequent shrinkage as the temperature falls to that of the surroundings.

Historically, vacuum mixing was introduced into the standard cement preparation methodology to reduce the porosity of the cement mantle as it was believed that pores act as crack propagation sites and therefore can significantly weaken the cement.^9^ In a review paper, Lewis reported that the majority of studies show that most cements, excluding Palacos R, show a significant increase in fatigue strength when mixed under vacuum compared to when mixed under non-vacuum conditions.^28^ In his own study Lewis reported that for a given cement, vacuum mixing significantly improves the fatigue performance.^29^ Vacuum mixing of bone cement reduces the porosity of the cement mantle created and therefore increases the amount of shrinkage from 2% to 5% for hand mixed cement^14^ to 3%–6% for vacuum mixed cement.^30^ Haas et al. reported that preventing the creation and expansion of pores within the cement through vacuum mixing may contribute to this increased extent of shrinkage.^14^ Bone cement does not form adhesive bonds but rather relies on mechanical interlock with bone trabeculae for fixation.^31,32^ Any deformation due to cement shrinkage after the cement has been formed to the bone may result in a reduction of the quality of the fixation between the bone and the bone cement. A key concern regarding shrinkage of bone cement is that any interstices created between cement and bone provides migration paths for wear debris from the articulating bearing to penetrate the interface and cause particulate-mediated osteolysis and subsequent aseptic loosening.^33^ A study that uses data from the Swedish national hip arthroplasty registry reports that risk of failure is initially increased due to vacuum mixing. However, the risk of failure gradually reduces and risk of revision when compared to open bowl hand mixing is lower after eight years.^34^

This paper focuses on cement shrinkage between the acetabulum and the cement mantle.

No studies were found that attempt to quantify the interstice created between the cement mantle and acetabulum during cemented acetabular implantation.

*In this study three questions were asked*:

Is there significant shrinkage of the bone cement mantle after cemented cup implantation?
Does vacuum mixing of the bone cement result in an increased bone cement mantle shrinkage?
Is the shrinkage uniform across the whole acetabulum?

## Materials

**Figure 1.**
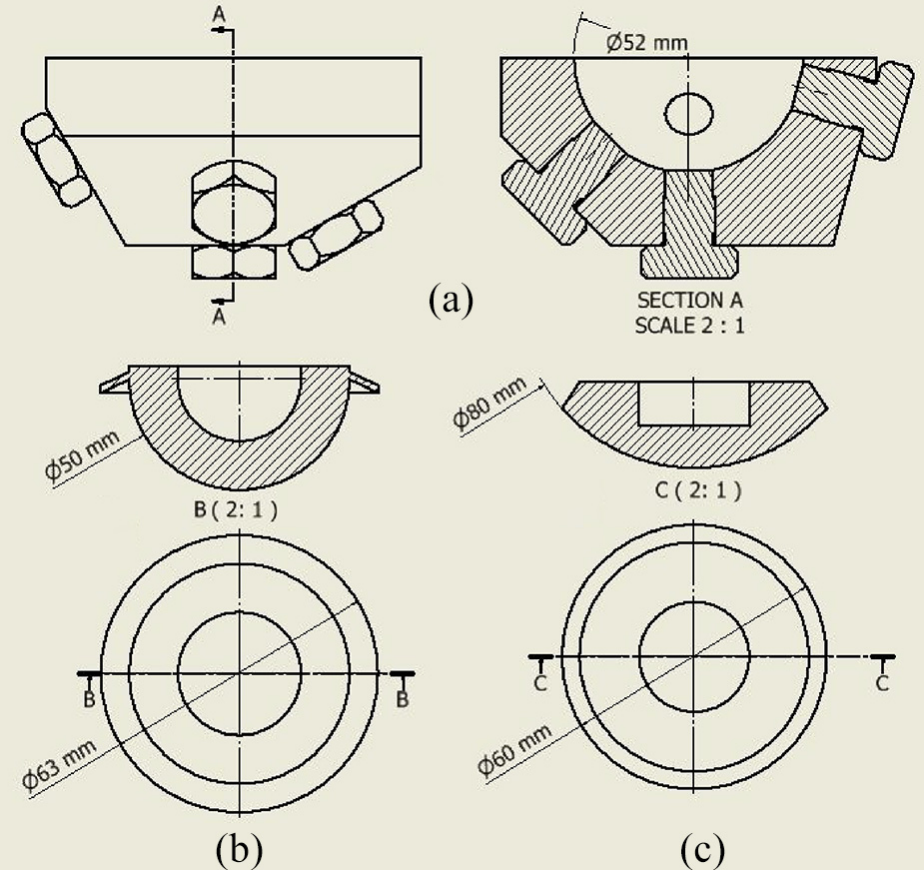
Engineering drawings with all relevant dimensions of the mock acetabulum: (a) acetabular cup (b), and the Depuy pressuriser (c).

The model acetabulum was manufactured from stainless steel 304. A 52 mm diameter hemispherical bore was reamed into the steel, a typical diameter to which the acetabulum is reamed (Figure 1(a)). A coordinate measuring machine (CMM) was used to measure the diameter of the cavity and confirmed that the bore diameter was within 0.01 mm of the expected value. The blanking bolts seen in Figure 1(a) filled holes used for pressure sensors in a separate experiment which is not reported here.

A flanged acetabular cup design was chosen and manufactured from HXLPE (highly-crosslinked polyethylene), which had an external diameter of 50 mm (Figure 1(b)).

A Depuy Smartseal acetabular pressuriser (DePuy, UK) was used for pressurisation of the cement. The pressuriser consists of a silicone spherical segment. When force is applied it is designed to seal off the acetabulum cavity with the cement still inside, thus pressurising the cement (Figure 1(c)).

**Figure 2.**
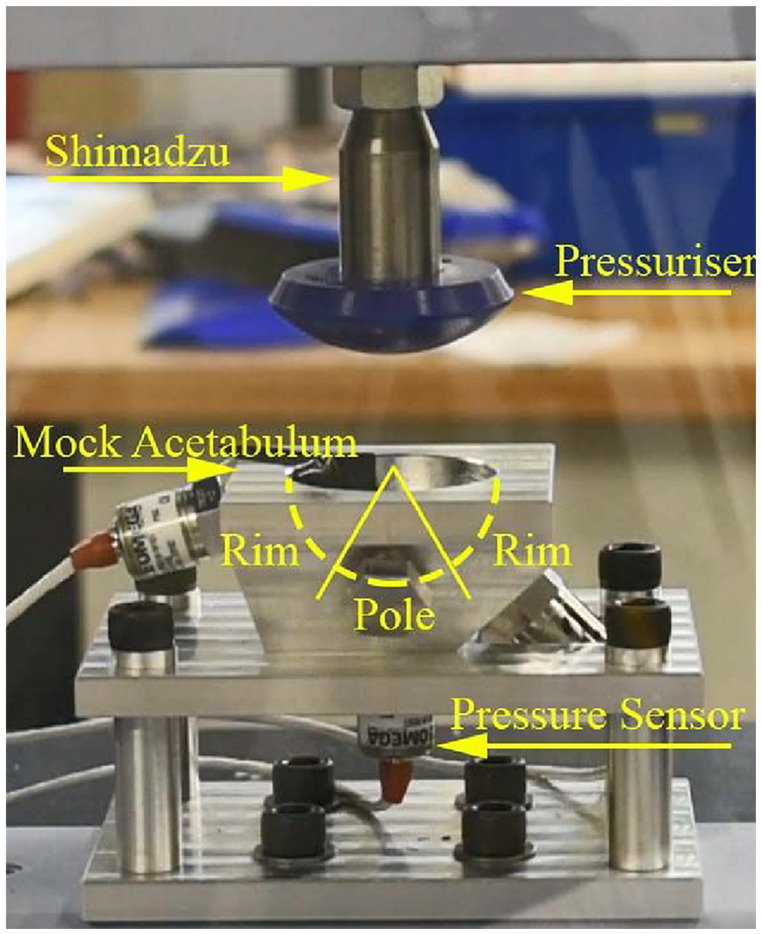
Mock acetabulum and Depuy pressuriser experimental set up with annotations and indication of rim and pole definitions.

The assembled rig, consisting of mock acetabulum and pressuriser (Figure 2) was mounted into a Shimadzu AGS-X, which was used to apply the force to the cup and pressuriser. The Shimadzu was fitted with a 1 kN load cell and was force controlled with a maximum stroke rate of 40 mm/min.

All equipment that was manufactured had a tolerance of ± 0.05 mm. Due to the design of the rig this means that the force will be applied within 0.25 mm of the centre of the acetabulum cavity.

## Methodology

All equipment used for the bone cement shrinkage experiments was kept at room temperature. Experiments were performed between 20.5°C and 23°C which is slightly outside the range defined by ISO 5833 (22°C–24°C) and the relative humidity was between 45% and 50% which is within ISO 5833 recommendations.^35^ Mould release spray (Silicone Mould Release Agent, Ambersil) was used to ensure that the cement mantle could be removed from the model acetabulum.

The bone cement was mixed by hand in an open bowl in five experiments and mixed under vacuum in five other experiments. For the open bowl, non-vacuum mixed conditions the cement was mixed in a glass bowl and mixed with an inert polyethylene spatula by hand at around 1 Hz until homogeneous and then left to rest until the cement past the doughing time.^35^ Doughing time is defined as the time at which fibers stop being created between a surgical glove and the cement mass after touching.^35^ For vacuum mixing, the cement was mixed using a Hivac Bowl (Summit Medical, UK) under a 0.4 bar (absolute) vacuum. The cement was mixed for 1 minute under vacuum then removed to test whether the cement had reach the doughing time.

**Figure 3.**
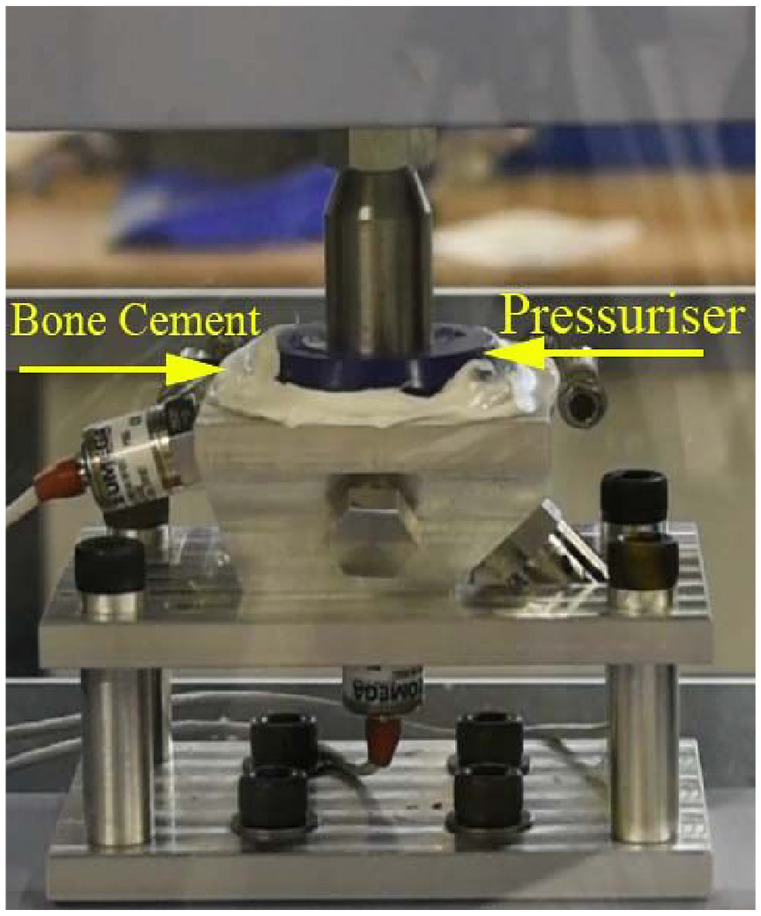
A force was applied to bone cement in acetabulum using 100 N force on Depuy pressuriser.

For both mixing conditions the cement was inserted into the model acetabulum when it had reached the doughing time and pressurised with a Depuy Smartseal pressuriser for 100 s at 100 N (Figure 3).^35^ In the experiment the orientation of the acetabulum was orthogonal to the direction of loading. Although during surgery the cup is implanted at an angle of 40° to the transverse plane of the body the force applied by the surgeon is still orthogonal to the plane of the cup face and therefore identical to the laboratory setup described here.

**Figure 4.**
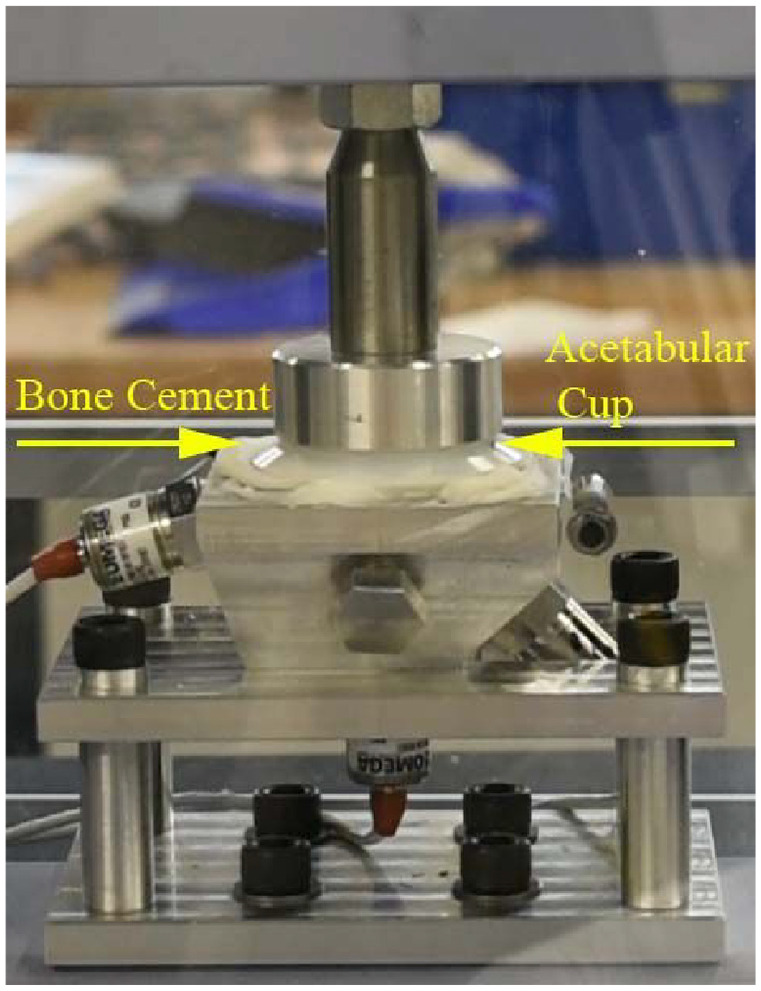
Pressure applied to the flanged acetabular cup using 50 N force.

After the pressuriser was removed, the acetabular cup was inserted into the cement mantle and a force of 50 N was applied. The force was removed after the cement had fully cured (Figure 4).

In the cited literature, the force applied during pressurisation and cup implantation were done by hand, this produced unrepeatable results. The forces used in this study were finalised after preliminary tests showed that the pressures produced at the surface of the acetabulum with this methodology were comparable to other studies in the literature.^36–39^ The timings were determined using the cement properties that can be found in the manual for the cement used.

**Figure 5.**
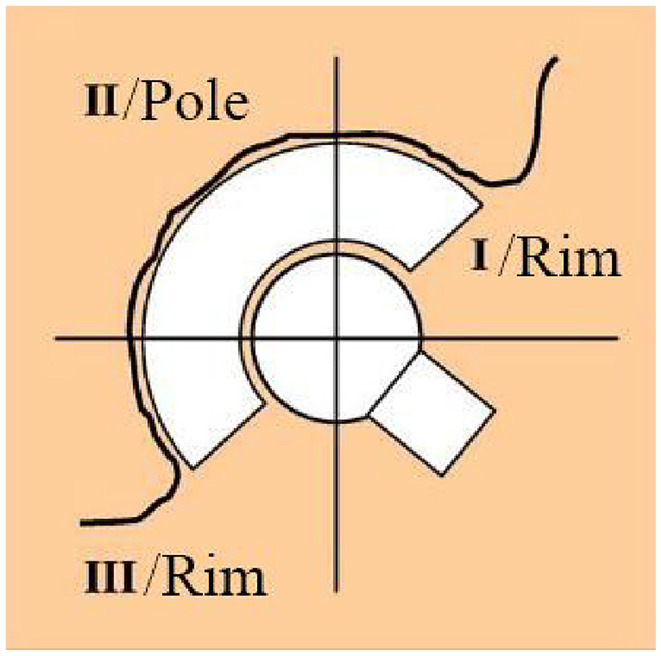
Acetabular zones as described by DeLee and Charnley.^3^

The experiment was concluded once the cement mantle had returned to room temperature, after this the cement mantle was removed from the acetabulum. The diameter of the resulting cement mantle was measured using a Mitutoyo Quickscope. The mantle was secured in the microscope and eight to fifteen coordinates were taken over the circumference of the mantle. A script was used to calculate the diameter of the mantle using the circumferential coordinates. This was repeated five times for each mantle. The precision of the coordinates taken were 0.0025 mm. This technique was then performed separately for the rim and the pole of the cement mantle to investigate whether the shrinkage was uniform. The rim was defined as the top 45° from the opening of the cavity (zone I and III) and the rim was all of the mantle below this (zones II) as this criterion was used by Delee and Charnley to describe the three zones of the acetabulum (Figure 5).

The measurement technique described was validated as follows: A spherical test piece of known diameter was measured, and the result was found to be within 0.05 mm of the true value. In addition, a coordinate measuring machine (CMM) was used to check the Quickscope measurements and this also found the result to be within 0.05 mm. Therefore, the Quickscope method was satisfactory for the level of accuracy required.

**Figure 6.**
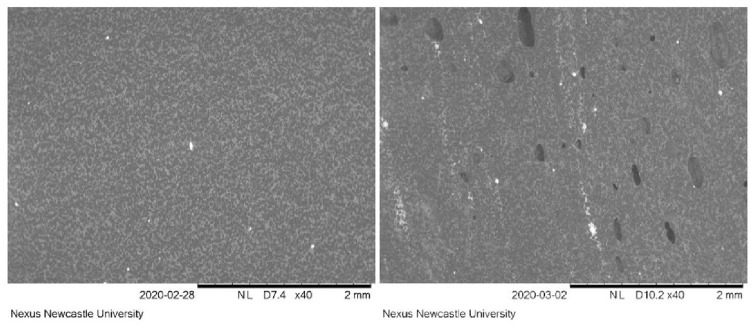
Representative SEM images taken for porosity calculations. Vacuum mixed cement on the left, non-vacuumed mixed cement on the right.

Two cement mantles from each mixing condition were sectioned so that the porosity and the mantle thickness could be measured. As the cement-bone interface was the focus of this study the internal diameter was not measured. The thickness was determined using a Vernier caliper; 10 measurements for the rim and the pole were taken and the results averaged. The porosity, reported as a ratio of pore area to total area, was determined using images taken on a Hitachi TM3030 scanning electron microscope (Hitachi, Japan) (SEM). Eight images were taken in total for each mantle: one in each quadrant of the sectioned area (Figure 6). This was repeated for the other half of the sectioned mantle.

A Ryan-Joiner test was used to see whether data were normally distributed; if so, a standard student *t*-test was used to determine whether there was a significant difference between compared variables shown in the table below (Table 1). If the data was not distributed normally, a Mann-Whitney test was used. A one sample t-test was used to determine whether the diameter was significantly different from the diameter of the model acetabulum. The results were considered significant if *p*≤ 0.05.

**Table 1.** Bone cement mantle dimensions for vacuum mixed and non-vacuum mixed cement for a 52 mm mock acetabulum and 50 mm HXLPE cup.

	Vacuum mixed	Hand mixed	Statistical difference?
Overall diameter (mm)	51.40 ± 0.042	51.61 ± 0.096	Y
Size of interstice (mm)	0.60 ± 0.09	0.39 ± 0.15	Y
Diameter at rim (mm)	51.31 ± 0.17	51.61 ± 0.10	Y
Diameter at pole (mm)	51.37 ± 0.11	51.56 ± 0.08	Y
Thickness at rim (mm)	4.94 ± 0.87	4.12 ± 1.07	Y*
Thickness at pole (mm)	10.09 ± 0.70	9.63 ± 0.34	N*
Porosity	0.0023 ± 0.0064	0.024 ± 0.023	Y*
Statistical difference? rim: overall diameter	N	N	
Statistical difference? pole: overall diameter	N*	N	
Statistical difference? rim : pole thickness	Y*	Y*	

All mean values with ± standard deviations (student *t*-test as standard, *for Mann–Whitney statistical tests).

## Results

Independent of whether the cement was mixed under vacuum or under non-vacuum conditions a significant interstice was created when PMMA bone cement was used to implant a 50 mm diameter HXLPE cup into a 52 mm diameter mock acetabulum. When the standard deviation and the precision of the manufactured acetabulum are included, the size of the interstice is dependent on the method of mixing: 0.60 mm ± 0.09 mm for vacuum mixed cement and 0.39 mm ± 0.15 for non-vacuum mixed cement. There was no significant difference between the magnitude of shrinkage at the rim and at the pole of the acetabulum for either mixing technique (Table 1).

The thickness at the pole of the cement mantles was larger than at the rim for every cement mantle (Table 1). Vacuum mixing resulted in a thicker mantle at the rim compared to non-vacuum mixing but there was no difference in the thickness at the pole because of the method of mixing (Table 1).

The porosity of the cement mantle also depended on the mixing method used (Table 1).

## Discussion

This study investigated shrinkage of the bone cement mantle after cemented cup implantation when the cement was mixed in vacuum and non-vacuum conditions. It also investigated whether shrinkage was uniform across the acetabulum. There was significant shrinkage of the bone cement mantle after acetabular cup implantation. Vacuum mixing significantly increased the magnitude of this shrinkage and was uniform across the whole acetabulum.

Although cemented metal on polyethylene hip replacements have the best survival rates of all fixation and material combinations at 1-year post-operation, 0.51% still fail. This means that 1730 of the 339,220 implants used to calculate this statistic will have failed within the first year.^1^ Some of these implants have radiolucent lines present on immediate post-operative radiographs indicating either a small interstice or a layer of soft tissue between the cement and bone.

This study found that when a 50 mm acetabular cup was implanted into a 52 mm reamed acetabulum, the outer diameter of cement mantle shrank by 0.39 mm ± 0.14 mm when the cement was mixed under non-vacuum conditions and by 0.60 ± 0.09 mm when the cement was mixed under vacuum. We hypothesise that this shrinkage may contribute to the development of an unstable interface. This is observed as radiolucent lines on immediate radiographs, to which the formation of fibrosis will be the hosts attempt to achieve stability. Ritter et al. found that when a radiolucent line was visible on the superior lateral area of the acetabulum immediately after surgery, 28.21% came loose. If no radiolucency was visible only 0.69% failed.^40^ Ranawat et al. reported that the state of the cement-bone interface of acetabular implants immediately post-operatively can be used to predict longevity of the implant.^6^ Hodgkinson el al. found that implants with a radiolucent line thicker than 1 mm that covered most of the cement-bone interface of cemented acetabular cups were all considered loose.^3^ Garcia-Cimbrelo et al. found that it was how much of the cement-bone interface was covered in a radiolucent line and not the thickness that determined whether the implant was likely to fail early.^41^ It should be noted that although these papers do not strictly qualify what ‘immediate’ means, it is unlikely that early radiolucent lines would be caused by osteolysis. Although our in vitro experimental cement shrinkage of 0.60 mm would likely contribute to the formation of immediate radiolucent lines in vivo, it is not likely they would be the sole cause. We also observed in vitro a significantly reduced shrinkage of the non-vacuum mixed cement mantles and this would imply smaller radiolucent lines if replicated in vivo. This hypothesis is also supported by the findings of Malchau et al. They found an increased risk of failure for the first 4 to 5 years after cemented total hip arthroplasty if the cement was mixed under vacuum when compared to if the cement was mixed in non-vacuum conditions.^34^ In addition to the shrinkage, interstice and the hypothetised increased risk of early loosening argument, the larger the interstice between the bone cement and the bone, the easier it is for particulate wear debris to migrate into this interface as described by Fick’s law of diffusion.^42^ Wear debris migration into the periprosthetic area between cement mantle and bone is a longer term process of loosening but may be synergistically linked to immediate post-operative cement shrinkage and resultant radiolucent lines. A study by Green et al. showed that particles 0.3 µm to 10 µm in diameter are the most biologically damaging; this is at least 39× smaller than the potential interstice created due to cement mass shrinkage.^33^

In this experiment, as a percentage diametric shrinkage non-vacuum mixed cement shrank by 0.75% and vacuum mixed cement shrank by 1.15%, due to irregularities in the geometry of the cement mantles it was not possible to reliably measure volumetric shrinkage. Gilbert et al. found that when Simplex P^TM^ was mixed under vacuum it had a volumetric shrinkage of 6.67% ± 0.40%, and 5.09% ± 0.50% when mixed by hand under non-vacuumed conditions.^30^ If it is assumed that shrinkage was uniform in all directions, then the estimated volumetric shrinkage is 3.41% for vacuumed mixed cement and 2.23% for hand mixed cement; however, as this study only measured shrinkage in one dimension the results should not be directly compared to literature. Despite this, the conclusions drawn are the same: Mixing the bone cement under non-vacuum conditions introduces many small pores into the cement. During polymerisation, these pores then grow to accommodate the shrinkage of bone cement caused by an increase in molecular density as MMA is converted to PMMA. If the cement is mixed under vacuum there are no points from which the cement shrinkage can occur within the cement mantle, therefore the outer dimensions of the cement mantle must change to accommodate the shrinkage.

The pole of all cement mantles was found to be thicker than the rim, this was due to the insertion of the cup being forced controlled rather than position controlled. Despite the discrepancy of the cement mantle thickness, the shrinkage of the cement was uniform across the entire mantle.

There are differences between this experimental in vitro study and the clinical in vivo setting. This study was designed to reduce confounding factors found in vivo in order to establish a baseline for the shrinkage behaviour. Further work can now be done to more closely model real in vivo behaviour. For example, the mock acetabulum was machined smooth whereas in vivo the cement will be pressurised into a partially porous acetabular bone bed with holes drilled in to aid interdigitation. Clinical practice assumes that if the cement is sufficiently interlocked with the bone a separation of cement and bone may be avoided. However, cement shrinkage will still occur and therefore if the bone cement and bone do not separate the bone trabeculae and the interdigitated cement will strain. We postulate this may cause damage to the interface leading to cracking and therefore radiolucent lines. In a clinical setting the cement will cool to 37°C, however, in this experiment it cooled to room temperature (20.5°C–23°C), this may alter the extent of the cement mantle shrinkage. The heating due to polymerisation would also cause changes to the dimensions of the mock acetabulum and therefore alter the dimensions of the resulting cement mantle. A steel manufacturer states that stainless steel has a thermal expansion coefficient of between 16 and 18 ×10^−6^/K.^43^ Lang found that fresh bovine phalanx bone had a linear axial thermal expansion coefficient of 89 ± 2 ×10^−6^/K.^44^ Using these values and the difference between room and body temperature the difference between the radius of the bone and the steel due to thermal expansion was calculated at less than 0.03 mm and therefore very small. The conductivity of the steel acetabulum was also not representative of bone. Sean et al. found that bovine cortical bone had a thermal conductivity of 0.58 ± 0.018 W/mK in the longitudinal direction, 0.53 ± 0.030 W/mK in the circumferential direction, and 0.54 ± 0.020 W/mK in the radial direction.^45^ The thermal conductivity of stainless steel 304 according to the manufacture is between 14 W/mK and 17 W/mK.^43^ This means less heat will be conducted away from the cement in a clinical setting and therefore the maximum temperature, the resulting thermal expansion and the consequential shrinkage upon cooling of the cement will be larger in vivo compared to this experiment. The geometry of this experiment was simplified to a pure spherical shape so the results would be reproducible – anatomically the rim of the acetabulum has many irregularities.

For this study the acetabulum model was dry, N’Diaye et al. found that PMMA bone cement experiences significant swelling due to water absorption, this swelling may reduce some of the shrinkage effects observed in this study.^46^ Ideally, during implantation the acetabulum should be dry to maximise interface strength and therefore the effects of swelling on the volume of the cement mantle will occur sometime after implantation.

The Shimadzu used to insert the acetabular cup was force controlled, this is partially representative of a real surgery in that the surgeon uses a combination of instinctive force control and estimated final position required; in our model this resulted in a slightly thicker cement mantle at the pole. Some preliminary studies were undertaken to try and position the cup so there was a uniform cement mantle, but due to the complex system dictating the viscosity of the cement, this methodology was not reliably repeatable.

Data for one type of cement has been presented in this current study. Future work should be done to investigate whether the results reported here apply to other types of cement.

The diameter of the acetabular cup used was 2 mm larger than that which is generally recommended by surgeons and manufactures. The effect of cup size on the size of the interstice created between the cement mantle and bone, and the pressure at the model acetabulum surface is currently being investigated.

Many of the limitations discussed above concern the differences between the clinical setting and this model. Therefore, the exact values for the diametric shrinkage may not be accurate. This in vitro study was an attempt to simplify the complex process that occur in vivo during cemented acetabular cup implantation and reduce confounding factors so that there could be confidence in the conclusions drawn.

It was previously known that PMMA bone cement shrinks during polymerisation, however, the magnitude of the shrinkage for vacuum and non-vacuum mixed cement mantles for cemented hip replacements was not known.

## Conclusion

This study found that the average size of the interstice created between the cement mantle and bone when a 50 mm diameter flanged acetabular cup is implanted into a 52 mm mock acetabulum is 0.39 mm ± 0.15 mm when the cement is mixed in non-vacuum conditions and 0.60 mm ± 0.09 mm when the cement is mixed under vacuum. This interstice is uniform across the cement mantle for each mixing methodology.

These findings offer an explanation for the increased risk of failure in the first 4 to 5 years after total hip arthroplasty when the cement is vacuum mixed compared to when it is mixed in non-vacuum conditions.^34^ It has been shown that immediate postoperative radiolucent lines are a good indicator for early failure of total hip arthroplasty.^6,40,41,47^ The cause of these lines has been thoroughly debated in the literature; the evidence presented here suggests that shrinkage of the bone cement is probably a contributing factor to these radiolucent lines and consequent early failure of cemented acetabular cups, especially when vacuum mixed. The cement mantle shrinkage may also provide a larger migration path for wear debris and increase the risk of periprosthetic osteolysis as a result.

Caution should be taken not to presume that the optimal cementing technique has been established. The best clinical evidence for determination of efficacy of operative techniques is arthroplasty registries, unfortunately many registries do not contain enough detail to make any conclusions regarding cement preparation techniques.
